# Mechanisms of the noxious inflammatory cycle in cystic fibrosis

**DOI:** 10.1186/1465-9921-10-23

**Published:** 2009-03-13

**Authors:** Mathilde Rottner, Jean-Marie Freyssinet, M Carmen Martínez

**Affiliations:** 1 INSERM U 770; Université Paris-Sud 11, Faculté de Médecine, Hôpital de Bicêtre, Le Kremlin-Bicêtre, France; 2 Université Louis Pasteur, Faculté de Médecine, Institut d'Hématologie et d'Immunologie, Strasbourg, France; 3 CNRS UMR 6214, INSERM U 771, Université d'Angers, Angers, France

## Abstract

Multiple evidences indicate that inflammation is an event occurring prior to infection in patients with cystic fibrosis. The self-perpetuating inflammatory cycle may play a pathogenic part in this disease. The role of the NF-κB pathway in enhanced production of inflammatory mediators is well documented. The pathophysiologic mechanisms through which the intrinsic inflammatory response develops remain unclear. The unfolded mutated protein cystic fibrosis transmembrane conductance regulator (CFTRΔF508), accounting for this pathology, is retained in the endoplasmic reticulum (ER), induces a stress, and modifies calcium homeostasis. Furthermore, CFTR is implicated in the transport of glutathione, the major antioxidant element in cells. CFTR mutations can alter redox homeostasis and induce an oxidative stress. The disturbance of the redox balance may evoke NF-κB activation and, in addition, promote apoptosis. In this review, we examine the hypotheses of the integrated pathogenic processes leading to the intrinsic inflammatory response in cystic fibrosis.

## Introduction

Cystic Fibrosis (CF) is the most common inherited autosomal recessive and lethal disease in caucasian population [1]. It is due to mutations in the product of the gene encoding the cystic fibrosis transmembrane conductance regulator (CFTR) [2]. This ATP-binding cassette (ABC) transporter is a membrane glycoprotein that functions not only as a cyclic AMP-regulated chloride channel in epithelial cells but also, as a reduced glutathione transporter [3,4]. Moreover, CFTR can regulate other channels such as the outwardly rectifying Cl^- ^channels (ORCC), epithelial Na^+ ^channels (ENaC), renal outer medullary K^+ ^channels (ROMK) or other inwardly rectifying K^+ ^channels [5]. It is expressed in various organs such as lung, pancreas, liver, gastrointestinal tract, and sweat glands [1].

More than 1,500 mutations of CFTR have been identified [6]. Mutations affect localization of CFTR at the apical plasma membrane and could interfere with its function and regulation. The most common mutation, deletion of phenylalanine at position 508 (CFTRΔF508), results in a misfolded protein that is retained in the endoplasmic reticulum (ER) [7]. The lack of functional CFTR at the plasma membrane correlates with impaired ionic balance and modification of cellular homeostasis. This pathological aspect is well documented and is characterized by thick secretions [8-11]. Thus, viscous and hyper-concentrated pancreatic secretions obstruct pancreatic ducts in CF patients accounting for the destruction of pancreas epithelium, which could promote inflammation [12,13]. Concerning the respiratory system, airway obstruction resulting from the thick mucus and reduced clearance of inhaled particles, including bacteria, promotes persistent infection and chronic inflammation [14] that are the major causes of death [15]. However, even in the absence of bacterial or viral pathogens, exacerbated inflammation has been reported in the respiratory tract of CF infants [16-18]. Hence, the origin of inflammation in CF has been a matter of debate and, recent data suggest that the retention of the misfolded protein could play a key role in the development and maintenance of the inflammatory response.

In the present review, we examine hypotheses of the coordinated and integrated pathophysiologic processes associated with CFTR defect retention in the ER that could lead to intrinsic inflammatory and apoptotic responses.

### Defects in CFTR and inflammatory responses

**Figure 1:**
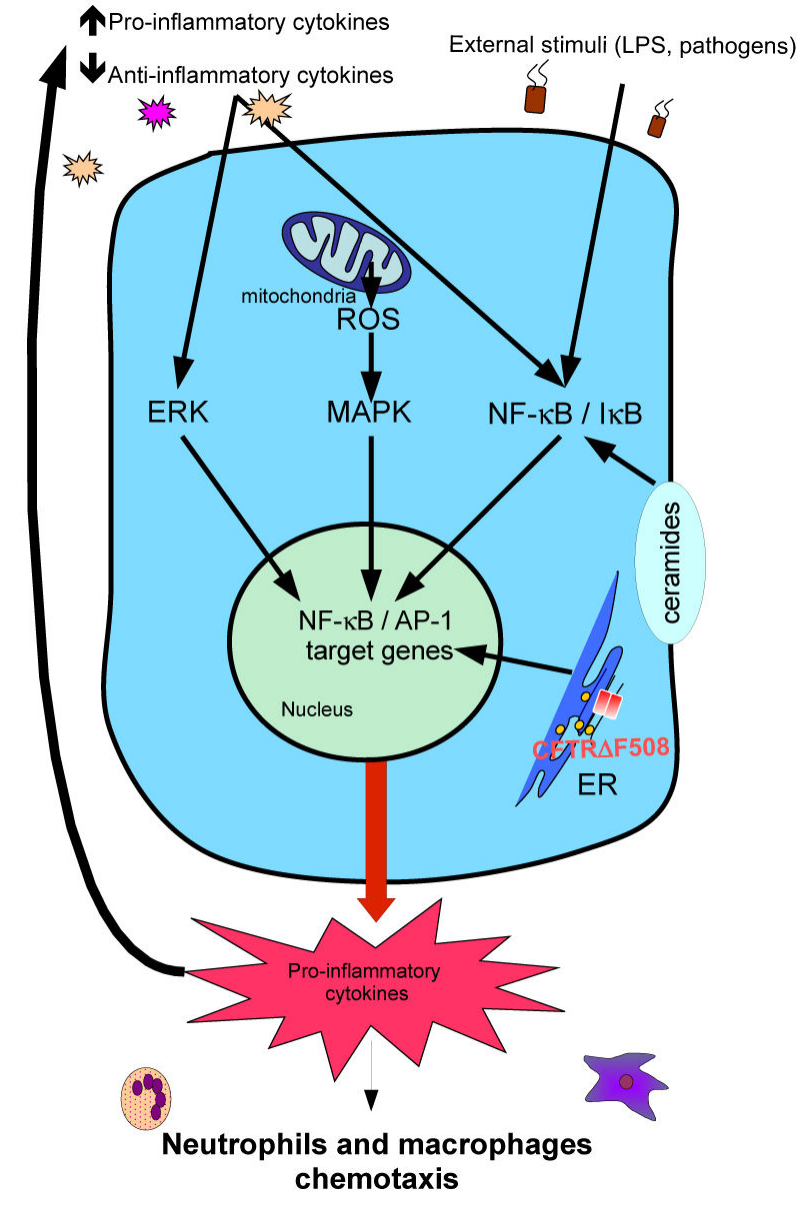
**Representation of the noxious inflammatory cycle in CF cell: Pro-inflammatory cytokines and external stimuli, such as pathogens, activate ERK pathway and promote dissociation of NF-κB/IκB complexe leading to NF-κB activation**. Retention of CFTRΔF508 in endoplasmic reticulum (ER), excessive production of reactive oxygen species (ROS) by mitochondria and alteration of ceramide levels leads to NF-κB activation, resulting in inflammation.

Although several studies have shown that CF and normal cells did not differ in both cytokine profile secretion and NF-κB activation [19,20], a large amount of data show that CF cells appear to produce excessive quantity of pro-inflammatory cytokines such as interleukin (IL)-8, IL-6 or RANTES [21-23]. Concretely, airway tract, and more particularly lungs, of CF patients present a high density of acute inflammatory cells, chiefly infiltrated neutrophils and macrophages, even in the absence of bacteria [24,25]. Moreover, airway infiltrated neutrophils are associated with high levels of pro-inflammatory mediators and cytokines like IL-8. In addition to a specific cytokine secretion profile, it has been observed that neutrophils from patients with CF show phenotype alteration(s) associated with functional and signaling changes, such as increased lipid raft assembly and increased levels of the cytoskeleton-associated phospho-Syk kinase [26]. Besides, blood and airway neutrophils in CF produce high basal levels of IL-8 that increase after lipopolysaccharide (LPS) treatment, suggesting an exaggerated basal pro-inflammatory cytokine secretion that could further augment under inflammatory conditions [27,28] (Figure 1). Co-cultures of polymorphonuclear (PMN) cells from CF and non-CF airways with CFTR-mutated and CFTR-corrected epithelial cell lines, respectively, have shown that PMN from CF airways and CFTR-mutated epithelial cell lines are responsible for pro-inflammatory cytokine production [29]. Furthermore, CFTR correction in epithelial cells did not allow return to levels of cytokine expression towards levels of normal cells, revealing an activated inflammatory intrinsic pathway in CF epithelial cells. In addition, a disturbance of the balance between inflammatory versus anti-inflammatory mediator production has also been described in CF cells [30,31]. Thus, the release of IL-10, the anti-inflammatory cytokine known to reduce IκB degradation (an inhibitor of NF-κB) [32], is reduced [33], whereas pro-inflammatory cytokines are over-expressed in association with an increase of both NF-κB and AP-1 activities that are under the control of IκB kinase and ERK signaling pathway (Figure 1). Moreover, pro-inflammatory cytokines, like IL-1β and FGF, promote activation of NF-κB and AP-1, participating in the perpetuation of the vicious cycle of inflammation [34]. Indeed, implication of NF-κB in inflammatory status in CF is well documented; AP-1 also appears to play an important role in inflammation [34,35]. However, the beneficial effect of miglustat [36], without affecting NF-κB and AP-1 status, is in favor of the implication of other transcription factors, notably ATF-6 implicated in the unfolded protein response (UPR) (see below) and Nrf-2 implicated in defense against oxidative stress [37].

In agreement with results obtained in cells cultured directly from uninfected CF tissues, Weber et al., [38] have found that defects of CFTR, in function of localization, contribute to endogenous activation of NF-κB, and consequently to the exaggerated production of the pro-inflammatory cytokine IL-8, even in the absence of bacteria. It should be noted that the link between CFTR defects and NF-κB activation must be strengthened during bacterial infection as described for *Pseudomonas aeruginosa *[39-41]. In addition, after LPS treatment a pro-inflammatory environment induces excessive NF-κB activation in CF macrophages, as above described for neutrophils [27,28], that is associated with a high production of pro-inflammatory cytokines [42,43]. This excessive production of cytokines results from CFTR dysfunction because the effect of LPS was also observed in healthy heterozygotes [44], suggesting that a single allelic CFTR mutation is sufficient for increase of the inflammatory response.

### CFTR and apoptosis

Contradictory data on the sensitivity of CF cells to apoptosis have been reported. Whereas intestinal epithelial CF cells show a higher fragmentation of DNA [45], suggesting an elevated susceptibility to programmed cell death, respiratory epithelial CF cells undergo delayed *Pseudomonas aeruginosa*-induced apoptosis [46]. Also, neutrophils from CF patients present a prolonged survival when compared to normal counterparts [47]. In agreement with these observations, an over-expression of the antiapoptotic protein Bcl-2 has been evidenced in CF patients [48]. By contrast, we have recently demonstrated [22] that CF epithelial cells from pancreas or trachea are more sensitive to apoptosis compared to normal cells. In addition, conditioned medium from apoptotic CF cells promotes apoptosis in normal cells, indicating that the release of mediators of inflammation during apoptosis is in turn able to evoke apoptosis [22]. Furthermore, macrophages from CF patients produce more TNF-α than their counterparts [49], and the increased secretion of Fas and FasLigand by CF epithelial cells [50], consolidate these observations. Increased susceptibility to apoptosis in epithelial cells and failed apoptosis in neutrophils would contribute to the self-perpetuating inflammatory cycle in CF. Independently of the susceptibility to apoptosis of CF cells, it has been shown that clearance of apoptotic cells is defective and that accumulation of such cells could contribute to ongoing inflammation in CF patients [51].

### CFTR and endoplasmic reticulum

ER has several specialized functions: (i) it is implicated in maturation, folding and transport of newly synthesized proteins; (ii) it is the site of biosynthesis for many lipids; and (iii) it acts as a calcium reservoir [52]. The ER contains intraluminal machinery composed of a large number of chaperone proteins implicated in the "quality control system" [53]. Proteins translocated into the ER lumen interact with chaperone proteins to acquire their conformation and then they are transported to the cell membrane or are secreted. Acquisition of conformation and formation of disulfide bonds needs resident chaperone proteins, high levels of calcium and an oxidative environment specific to the ER [54]. Accumulation of misfolded proteins, alterations of calcium homeostasis, inflammation or hypoxia, causing perturbations in environment, lead to disrupted ER function, also referred as to "ER stress" [55]. The most common mutation of CFTR produces an incorrectly folded protein, CFTRΔF508, which is present within the ER, and accumulates in the ER-Golgi intermediate compartment [56] causing an ER stress. In addition, in ER CFTRΔF508 interacts with calcium-dependent chaperones, modifying calcium homeostasis [57] and generating further stress [58].

**Figure 2:**
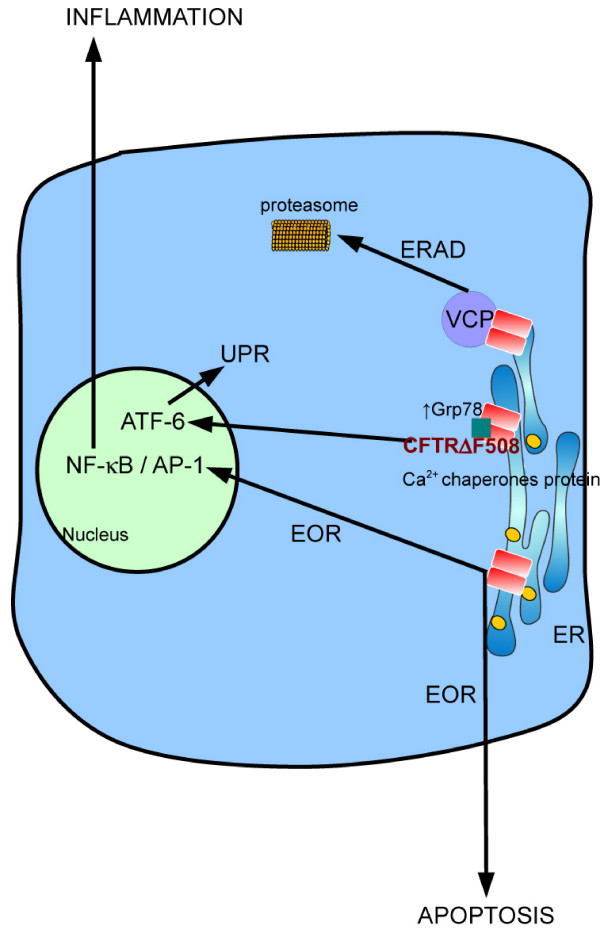
**Involvement of misfolded CFTRΔF508 in endoplasmic reticulum (ER) stress in CF cell: Retention of CFTRΔF508 is associated with an increase of Ca^2+ ^concentration in the lumen of ER and with the interaction with chaperone proteins**. This induces activation of ERAD in order to degrade misfolded CFTRΔF508 by proteasome. In addition, increase of Grp78 induces activation of the UPR via the ATF-6. Whether ERAD and UPR are not sufficient to restore normal cellular parameters, EOR is activated resulting in apoptosis induction.

Under ER stress conditions, three different pathways are activated in order to reduce the synthesis of new proteins and to increase degradation of uncorrected proteins (Figure 2): (i) the UPR that leads to a reduction of protein synthesis and transcription of chaperone target genes, (ii) the ER-associated degradation (ERAD) in order to eliminate misfolded proteins by proteasome, and (iii) in case these responses are unsuccessful, the ER-overload response (EOR) leading to apoptosis [59]. ER stress generated by misfolded CFTRΔF508 protein activates the UPR and the over-expression of several ER resident chaperones such as Grp78 [60], resulting in decreased CFTR expression [61,62]. In addition, in CFTRΔF508 cells, Kerbiriou et al. [60] have described an increase of ATF6 form, an initiator of UPR that is accompanied with its translocation into the nuclei. Using cells expressing different mutations of CFTR, Weber et al. [38] have demonstrated that accumulation of CFTR in the ER contributes to the endogenous activation of NF-κB. Also, Verhaege et al. [34] have shown that activation of NF-κB and AP-1 transcription factors are dependent on the ER sequestration of the misfolded CFTRΔF508 protein.

ERAD, a component of a coordinated cellular response to ER stress, can be activated and induces protein removal from the ER and degradation in the cytosol, via the ubiquitin-proteasome system. Thus, after being selected by quality control system, the ubiquitin proteasome system interacts with unfolded ER proteins and cofactors that permit their translocation from ER to cytosol, where they are polyubiquitynated by ubiquitin ligase system, and then degraded by cytosolic proteasome system. Among the misfolded proteins processed by ERAD and the ubiquitin proteasome system, CFTRΔF508 is detected in the cytosol and within the ER membrane by the ERAD components, and finally, it can be degraded by proteasome [63,64]. In particular, in CF bronchial epithelial cells from CFTRΔF508 homozygous patients, the p97/valosin-containing protein (VCP), which is an integral component of ERAD, is over-expressed compared with non-CF bronchial epithelial cells. Moreover, inhibition of VCP induces the rescue of CFTRΔF508 protein to the plasma membrane that is associated with the diminution of NF-κB activation and the reduction of IL-8 secretion. The fact that the ERAD system, or at least some of the components, is up-regulated in CF could explain, in part, the exaggerated NF-κB activation in cells presenting CFTR defects [65].

When UPR and ERAD signaling pathways are not sufficient to restore normal cell parameters, cells could activate EOR in order to induce apoptosis [59]. It has been described that, independently of UPR activation, the accumulation of unfolded proteins in the ER membranes provides an NF-κB-activating stimulus [66]. Several messengers have been proposed as signals from the ER for NF-κB activation, such as the efflux of calcium or the reactive oxygen species (ROS) [67]. It is possible that the constitutive NF-κB activation observed in CF cells could be the consequence of EOR in response to CFTR accumulation in the ER membranes as described by Knorre and colleagues [68]. Exaggerated NF-κB activation, apoptosis and pro-inflammatory mediator productions have also been described in CF pancreatic and tracheal cells [22] suggesting that EOR may be implicated in theses events.

Altogether, prevention of the retention of CFTR in the ER and its rescue at the plasma membrane may be a key element in CF pathogenesis, and CFTRΔF508 correction based on the activation of different ER-linked systems can represent new approaches of this disease [69-71].

### CFTR and calcium homeostasis

In agreement with the observations that ER plays a major role in the CF pathology, it has been shown that calcium homeostasis is altered in cells presenting CFTR mutations. Antigny et al. [57] have shown that the release of calcium from ER stores by agonists, such as histamine or ATP, is increased in CF cells. More important, the rescue of CFTRΔF508 at the plasma membrane by incubating cells at low temperature (27°C) restored calcium mobilization at similar levels than those measured in normal cells.

In CF human airway epithelia, other studies have described a higher calcium mobilization in response of G protein-coupled receptors to nucleotides or bradykinin, suggesting that the apical ER/calcium store compartment is expanded [72]. However, these authors propose that the increase in ER volume could be attributable to airway infection and inflammation rather than the intrinsic CFTR defects since in the absence of infection or after long term culturing CF cells, ER expansion is abolished. In addition, same authors have established a relationship between inflammation and ER expansion [73]. Indeed, the increase in IL-8 secretion induced after bradykinin treatment was mediated by an increased calcium mobilization consecutive to ER expansion. The link between inflammation and calcium mobilization has been confirmed by the work from Tabary et al. [74]. Using a model of individual living airway epithelial cell monitoring, they have shown that inflammatory mediators such as IL-1β are able to induce an increased calcium release, which is accompanied by activation of NF-κB in CF cells. Depletion of calcium stores from ER or inhibition of NF-κB activation leads to a decrease in calcium responses suggesting that inflammatory cytokines regulate calcium handling in CF cells, and in turn, calcium mobilization controls NF-κB activation contributing to a noxious cycle. In this context, the most common pathogens in CF, *Pseudomonas aeruginosa *and *Staphylococcus aureus*, promote an increase of intracellular calcium concentration in CF cells that lead to NF-κB activation and proinflammatory cytokine production via ERK1/2 and p38 pathways [75].

Other evidences suggest that the excess of calcium sequestration in ER may contribute to the abnormal trafficking of CFTRΔF508. Indeed, it has been demonstrated that the decrease and the maintenance of low calcium levels in ER, by using SERCA inhibitors, prevent the interaction between CFTRΔF508 and chaperones and thus this restores CFTRΔF508 at the plasma membrane [76,77].

In addition to ER expansion, mitochondria from CF lymphocytes display calcium accumulation [78], that could be explained by the higher activities of several enzymes involved in the cell energy metabolism such as NADH oxidase, NADH- and succinate-cytochrome c reductases [79].

### CFTR and oxidative stress

When the balance between antioxidants and oxidants is no longer able to prevent the alteration of physiological functions, oxidative stress takes place. In airways in particular, ROS, a general term for a number of various and highly reactive oxygen derived ions or molecules, including both radicals and non-radicals, are the main oxidant species. ROS display beneficial and deleterious effects, depending of their concentration; an increase occurs when cells present an imbalance between the production and the neutralization of free radicals by antioxidant defense systems. Thus, ROS excess has been described to promote inflammatory gene transcription [80]. In addition, the interaction of ROS with nitric oxide (NO), resulting in reactive nitrogen species formation, can further enhance their potential deleterious effects [81,82]. Among the effects due to excessive production of ROS, the oxidation of macromolecules causes irreversible damage in molecular targets like proteins, DNA and lipids [83].

### CFTR and GSH

Glutathione, an ubiquitous tripeptide, is one of the most important antioxidant molecules. Glutathione exists in reduced monomeric (GSH) and oxidized dimeric forms (GSSG). GSH is found in extracellular fluids, in lung and in cells at high concentrations of its reduced form. Extracellular GSH neutralizes free radicals produced by neutrophils during inflammation [84]. Taking into consideration that CFTR permits the transport of GSH between cells and apical extracellular media [85], it is reasonable to imagine that intracellular GSH content may be altered in CF. In this respect, contradictory data on GSH content in lung fluid of CF patients have been reported (for review, see [86]). Indeed, in epithelial lining fluids, like plasma, the concentration of GSH, but not GSSG, is reduced compared with normal subjects [25]. The level of GSH in plasma of CF patients is also reduced suggesting that GSH deficiency is not limited to the site of inflammation but is rather systemic [25]. Gao and collaborators [84] have shown that, in cell lines expressing CFTR mutations or transfected with normal CFTR, GSH efflux is lower in the former indicating an abnormal transport of GSH associated with a defective CFTR. Interestingly, it has been reported that nebulized buffered GSH or the combination of oral GSH and inhaled buffered GSSG attenuates CF disease [87,88], suggesting that new approaches against altered cellular redox status may represent potential treatments of CF. Not only cellular content of GSH is altered in CF, low mitochondria GSH levels have also been described in both lung from CFTR-deficient mice and human lung epithelial cells lines expressing CFTR mutations [89]. Because GSH is known to inhibit IκBα degradation [90,91], low levels of GSH in CF cells may promote NF-κB activation and participate in the maintenance of inflammation. By contrast, Jungas et al. [92] have measured an increase of intracellular GSH levels in epithelial cells (HeLa) transfected with CFTRΔF508 compared to wild-type cells, and this is associated with a defective apoptosis in CF cells probably due to a slower GSH depletion. In agreement with this work, Day et al. [93] have shown an increase of GSSG in epithelial lining fluid from CFTR-deficient mice after *Pseudomonas aeruginosa *infection resulting in a reduction of the GSH/GSSG ratio indicating an increased oxidative stress.

### CFTR and reactive oxygen species

**Figure 3:**
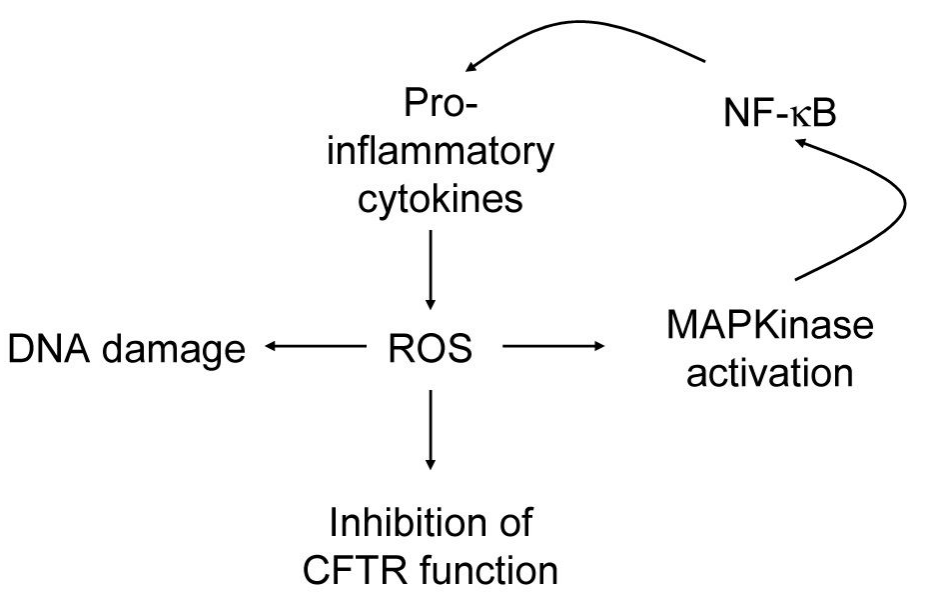
**Reactive oxygen species (ROS) affect DNA integrity, CFTR function and can participate in the noxious cycle of inflammation in CF cells**.

Elevated markers of oxidative stress, like lipid hydroperoxidation and protein oxidation, have been measured in plasma from CF patients and are associated with diminished concentration of plasma antioxidants [94,95]. Thus, elevated lipid peroxidation could be associated with pulmonary dysfunction leading to damage of structural membranes. An elevation of urine concentration of a marker of ROS-induced DNA damage, 8-hydroxydeoxyguanosine, has also been described in CF patients, suggesting that in CF an increased susceptibility to oxidative-induced DNA damage may explain the further incidence of malignancy [96] (Figure 3).

In epithelial lining fluid in lung of CFTR-deficient mice, Velsor et al. [89] have shown an increase of markers of oxidative stress related to lipid and DNA oxidation. Furthermore, in a CF epithelial cell line, they have reported high intracellular levels of hydrogen peroxide, reflecting oxidative stress. Moreover, mitochondrial levels of ROS (superoxide anion and hydrogen peroxide) are enhanced in CFTR-deficient cell line [97]. Altogether, these results indicate that elevated levels of mitochondrial and cellular ROS are associated with a CFTR-deficient state. The effects of ROS may be double in CF, on the one hand, it is known that cellular stress induced by ROS inhibits CFTR maturation, levels and function [58] (Figure 3). On the other hand, an increase of ROS leads to MAPK signaling pathway activation [83]. Because this cascade is known to regulate pro-inflammatory gene expression in CF cells [34], it is reasonable to hypothesize that ROS are implicated in the initiation or/and the maintenance of the inflammatory state in CFTR deficiency. In addition, an excessive production of pro-inflammatory cytokines could increase ROS production [98] perpetuating the vicious circle of inflammation in CF (Figure 3).

Defective neutralization of ROS can also elicit oxidative stress. Three superoxide dismutases (SOD) have been described in mammals, Cu/Zn-SOD or SOD1, Mn-SOD or SOD2, and extracellular-SOD or SOD3 (for review see [99]). These enzymes are implicated in decreasing superoxide anion levels that damage cells at excessive concentration [100]. Alterations in the expression or/and activity of SODs have been described in several pathologies such as amyotrophic lateral sclerosis for SOD1 [101], cardiomyopathies for SOD2 [102] and lung diseases for SOD3 [103]. Indeed, SOD3 is highly expressed in lungs and is associated with decreased recruitment of neutrophils, suggesting an important role in regulating pulmonary inflammation [99]. Although no direct evidence has shown the involvement of SOD3 in CF, the fact that it is highly expressed in airways raises the possibility that it may play a role in CF. In addition, pro-inflammatory cytokines increase SOD3 expression, in culture and in animal models of lung injury [104,105].

In agreement with these studies, we have observed a diminution of the expression of three isoforms of SOD, in pancreatic and tracheal CF cells compared to their respective controls. In addition, direct measurements of superoxide anion production by electronic paramagnetic resonance correlate with the diminution of SOD expression suggesting an adaptive response to oxidative stress, in CF cells (unpublished results).

Taken together, deficiency in antioxidant systems seems to be associated with all CFTR mutations. Thus, in CF patients, inadequate antioxidant defenses are associated with the elevated oxidative stress, which contributes to the decline of pulmonary function.

### CFTR and NO

NO plays a dual role under inflammatory conditions. On the one hand, NO acts as a bronchodilator molecule, is able to modulate immune responses, possesses antimicrobial activities and acts as an important signaling molecule. One the other hand, reactions of NO with ROS can originate intermediate reactive species that have deleterious properties. Three different isoforms of NO synthase are expressed in normal lung tissue but only one, iNOS, leads to production of NO that has antimicrobial properties by reducing *Pseudomonas aeruginosa *adherence. Whereas expression of iNOS is upregulated in inflammatory diseases [106], expression of this enzyme is decreased in CF [107,108], which may explain, at least partially, the chronic airway infection. Although sites and techniques for NO measurement are subjects at debate, patients with CF have lower NO levels, certainly in parallel with low expression of iNOS, but present high levels of peroxynitrite, nitrite and nitrates [109-114]. A possible explanation has been proposed by Jones et al. [115], suggesting the formation of nitrates and other NO metabolites by reaction with superoxide anion, and then NO levels are reduced. In addition, there is increasing evidence that decreased NO formation contributes to airway obstruction in CF [111,116-118]. Interestingly, low NO levels are correlated to both pancreatic insufficiency and chronic *Pseudomnas aeruginosa *infection, suggesting that airway NO could be associated with genotype and could be considered a risk factor for infection [111]. S-Nitrosothiols, NO adducts that can mediate several effects of NO, are also decreased in CF airways [119]. Thus, treatment with S-nitrosothiols is able to increase expression, maturation and function of both wild-type and mutated CFTR [120]. Moreover, low levels of S-nitrosoglutathione, a physiological NO donor that acts as an innate-immune mediator, can enhance the deleterious effects of *Pseudomonas aeruginosa *by affecting its metabolism [118]. Furthermore, treatment of CF homozygote ΔF508 mice with LPS has no effect on NO production, suggesting that decreased expression of iNOS could contribute to chronic infection of the airways [119].

### CFTR and ceramides

Ceramides are sphingolipids, located in vesicles and cell intermembranes. They are generated by hydrolysis of sphingomyelin by acid sphingomyelinase (ASM) or by de novo synthesis by ceramide synthase. They are degraded by ceramidase to form fatty acid and sphingosin. Ceramides play crucial rules in signaling pathways. Recent data suggest that ceramides may be involved in the pathogenesis of CF. For instance, infection through *Pseudomonas aeruginosa *favors interaction with rafts and activates ASM, the latter translocates from intracellular vesicles to the extracellular leaflet of the cell plasma membrane and generates ceramides. Then, ceramides induce rafts fusion to form membrane platforms with receptors and molecule clusters, like CD95 and CFTR, initiating signalization [121], leading to cell death [48], regulation of NF-κB pathway [122], and expression of pro-inflammatory cytokines (Figure 1). Therefore, ceramides may represent an important target for understanding and treating CF. Like for apoptosis, levels of ceramides in CF cells are a matter of debate. Vilela et al. [48] have shown a decreased ceramide production in CFTRΔF508 lung epithelial cells after treatment with TNF-α. For these authors, low levels of ceramide production is due to high GSH levels that inhibit ASM. This results in an exaggerated inflammation due to absence of NF-κB inhibition by ceramides. Recently, Teichgräber and colleagues [122] observed a ceramide accumulation, age-dependent, in ciliated respiratory, nasal epithelial cells, submucosal glands, and macrophages from CFTR-deficient mice. In addition, accumulation of ceramides, probably through an imbalance between generation and degradation, is associated with constitutive pulmonary inflammation and epithelial cell death. Most interestingly, pharmacological inhibition of ASM, in CFTR-deficient mice concomitantly normalizes ceramide production and decreases pathological parameters [122]. Altogether, these results and those of Vilela et al. [48] demonstrate a modification of ceramide homeostasis in CF associated with an exaggerated inflammatory response to various stress inducers.

## Conclusion and Perspectives

Intrinsic inflammation in CF, in the absence of pathogens, has multiple origins that make it difficult to resolve. In addition, contradictory data on apoptosis in CF highlight the need of further studies in order to explore the status of programmed cell death in this pathology. As detailed above, whether ER stress remains sustained, as this occurs with CFTRΔF508 retention, cells initiate programmed death, and this could explain the activation of NF-κB-associated pathways and inflammation observed in CF. Although gene therapy may symbolize a real solution for the disease, the enthusiasm has been rapidly tempered in view of the difficulties associated with the use of viral and non-viral vectors, as well as the complexity of the pathways controlling CFTR function. In this line, carrier proteins delivering pharmacological chaperones may be considered good candidates to new therapies leading to correction of mutated CFTR [123]. However, very recently, several pathways, up to date not linked to CFTR deficiency, have been proposed to modulate inflammatory response in CF and may represent new therapeutic approaches.

Among these pathways, the peroxisome proliferator-activated receptor-γ (PPARγ) may be considered because of its ability to participate in the transcription of various genes involved in the regulation of the inflammatory response [124]. In this respect, it has been recently described that up-regulation of tissue transglutaminase may account for the reduction of PPARγ expression in CFTR-defective cell lines, and in this way, tissue transglutaminase inhibition could regulate inflammation in CF cells [125]. In addition, these authors have shown that increase of intracellular calcium and excess of oxidative stress modulate tissue transglutaminase activity, reinforcing the notion that both calcium and ROS are implicated in the inflammatory response in CF. In the same way, Perez et al. [126] have shown that PPARγ agonists reduce cytokine secretion *in vitro *and airway inflammation in response to *Pseudomonas aeroginosa *in CF mice.

Natural products, mainly from vegetables, may also represent potential therapy in CF. In particular, flavonoids and other bioactive compounds, with a large spectrum of biological effects such as anti-oxidant potential, could affect different stages of CF pathogenesis. For instance, several plant extracts have been found able to decrease secretion of inflammatory cytokines from CF cells [127]. Others correct defective electrolyte transport in CF airways acting in parallel on second messengers and channel activities [128]. Curcumin has also been described as a corrector of the CFTR defect by eliciting increased CFTR traffic towards the plasma membrane [129].

Nevertheless, until gene therapy is completely successful, inflammation, in addition to elimination of infection mucus, remains the main target in the treatment of CF. To improve the effects of the different therapies, a better knowledge of the intracellular and molecular mechanisms involved in the regulation of CFTR traffic and function seems essential for correction of mutated CFTR at different stages of impaired functions.

## Competing interests

The authors declare that they have no competing interests.

## Authors' contributions

MR participated in the design of the study and wrote all chapters; JMF contributed to the final revision of the manuscript; MCM designed and wrote the paper, participated in the revision and editing of the manuscript. All authors read and approved the final manuscript.
